# Systematic review of single-incision versus conventional multiport laparoscopic surgery for sigmoid colon and rectal cancer

**DOI:** 10.1186/s12957-018-1521-4

**Published:** 2018-11-10

**Authors:** Xin Liu, Ji-bin Li, Gang Shi, Rui Guo, Rui Zhang

**Affiliations:** Department of Colorectal Surgery, Cancer Hospital of China Medical University, Liaoning Cancer Hospital and Institute, No 44 Xiaoheyan Road, Dadong District, Shenyang, 110042 Liaoning Province People’s Republic of China

**Keywords:** Single-incision, Meta-analysis, Laparoscopic surgery, Sigmoid colon and rectal cancer

## Abstract

**Objectives:**

To explore whether single-incision laparoscopic surgery (SILS) has the better short-term clinical and pathological outcomes than conventional multiport laparoscopic surgery (CLS) for sigmoid colon and rectal cancer.

**Methods:**

A literature investigation of MEDLINE, PubMed, Ovid, Embase, Cochrane Library, Web of Science, Chinese National Knowledge Infrastructure (CNKI), Chinese Biological Medicine (CBM), and Wanfang databases for relevant researches was performed. Fixed effects and random effects models were used to calculate the corresponding outcomes. Standardized mean difference and risk ratio were calculated for continuous and dichotomous variables separately.

**Results:**

Nine clinical controlled trials were composed of two randomized clinical trials and seven non-randomized clinical trials with a total of 829 patients. Two hundred ninety-nine (36.1%) patients underwent SILS, and 530 (63.9%) patients underwent CLS. The meta-analysis showed that SILS had more lymph node resection (SMD − 0.25, 95% CI − 0.50 to − 0.002) and less defecation time (SMD − 0.46, 95% CI − 0.75 to − 0.17), exhaust time (SMD − 0.46, 95% CI − 0.75 to − 0.18), and hospital stay (SMD − 0.30, 95% CI − 0.45 to − 0.15 than CLS. SILS was also accompanied with shorter incision length (SMD − 2.46, 95% CI − 4.02 to − 0.90), less pain score (SMD − 0.56, 95% CI − 0.91 to − 0.21), and lower complication rate (RR 0.66, 95% CI 0.47 to 0.91). Blood loss, operative time, distal margin, conversion rate, anastomotic fistula, readmission, local recurrence, and distant metastasis showed no statistical differences in two groups. In all subgroup analysis, SILS also had advantages of incision length, operative time, defecation time, exhaust time, and hospitalization time than CLS.

**Conclusion:**

SILS could be a more safe and reliable surgical technique than CLS for sigmoid colon and rectal cancer. However, further high-quality studies between these two techniques need to be further developed.

## Background

Conventional multiport laparoscopy (CLS) is increasingly being used in colorectal surgery. CLS had the advantages of faster recovery, reduced morbidity, and blood loss, but also had incision-related complications. Since single-incision laparoscopic surgery (SILS) was developed in 2008, incision-related complications of hemorrhage, incision rupture, and organ damage have been greatly reduced [1–3]. There were different opinions about the clinical efficacy between SILS and CLS.

Several published meta-analyses evaluating SLIS versus CLS have shown that short-term clinical and oncological outcomes of SILS are better than that of CLS [4]. Li et al. had very fully confirmed that SILS had less blood loss, shorter incision length, shorter and hospital stay but longer operative time for colorectal disease [5]. However, laparoscopic sigmoid and rectal surgery based on these two techniques has rarely been studied by meta-analysis. Here we comprehensively compared the clinical outcomes of two techniques for treatment of sigmoid and rectal cancer.

## Methods

### Literature search

We had systematically collected useful studies from MEDLINE, PubMed, Embase, Cochrane Library, and Wanfang from 2010 to 2018. Search terms included “laparoscopy,” “single incision,” “single port,” “single site,” “SILS,” “CLS,” “sigmoid cancer,” “rectal cancer,” and “TME (total mesenteric resection).” Manual searches of references from relevant articles were performed when necessary. We increased the scope of the research by “related articles” option. Included studies were English or Chinese human researches with the abstracts, scope, and reference checked.

### Eligibility criteria

One hundred seventy-nine studies searched from the Internet were separately screened by three investigators according to the following inclusion criteria: (1) comparing the outcomes of SILS versus CLS for sigmoid or rectal cancer, (2) one outcome mentioned at least, and (3) randomized clinical trials (RCTs), non-randomized controlled trail (NRCTs), or comparative observational (cohort and case-control) studies.

Additionally, the exclusion criteria were as follows: (1) related research was not about sigmoid colon or rectal disease, (2) the relevant data were not specifically reported, and (3) conference articles, case, letters, and other unqualified articles.

### Types of interventions

Laparoscopic surgery was performed through a laparoscope with special instruments by a small incision length. CLS always had three or more ports, while SLIS had only one port for surgery.

### Outcome of interest

We used the following results to compare SILS and CLS: (1) intraoperative data based on operative time, incision length, amount of bleeding, conversion, lymph node resection, and distal surgical edge; (2) postoperative data including complication, anastomotic fistula rate, defecation time, exhaust time, pain score, and hospitalization time; and (3) short-term follow-up data including readmission, local recurrence, and distant metastasis. Subgroup analysis of tumor location (sigmoid colon and rectal cancer), region (eastern and western), and language (Chinese and English) were conducted.

### Data extraction

The literatures were searched according to the above criteria by two reviewers independently. The following data were collected: (1) the first author(s) and publication data, (2) the study area, (3) the characteristics of patients in each group, and (4) the quality of the study. A third reviewer was introduced to resolve all disagreements about the articles until a consensus was reached.

We contacted the authors of all studies with incomplete data but did not get any additional information. As referred to in the missing data of means and SDs, we calculated them based on medians and ranges according to availability [6, 7].

### Risk of bias evaluation

Two RCT qualities were assessed by the Cochrane Reviewers’ Handbook with the Jadad score in three metrics: randomization, double blindness, and control.

The quality of NRCTs was assessed with the Newcastle-Ottawa Scale from three aspects: patient selection, confirmation of exposure, and comparability of both groups [8].

### Statistical analysis

This study followed the Preferred Reporting Items for Systematic reviews and Meta-Analysis (PRISMA) guidelines. We used Stata 11.0 to compare two groups by standardized mean differences (SMD) with 95% confidence intervals (95% CIs) for continuous data and relative risks (ORs or RRs) with 95% CIs for dichotomous outcomes. The statistical heterogeneity was estimated by *I*^2^ statistic and *χ*^2^ test.

When *I*^2^ > 50% and *I*^2^ < 50%, random effects and fixed effects models were utilized separately. *P* < 0.05 indicated statistical differences. Begg’s test was used to evaluate publication bias. Sensitivity analyses were conducted by sequentially excluding studies one by one to decrease the impact of single study.

## Results

### Study characteristics

**Fig. 1:**
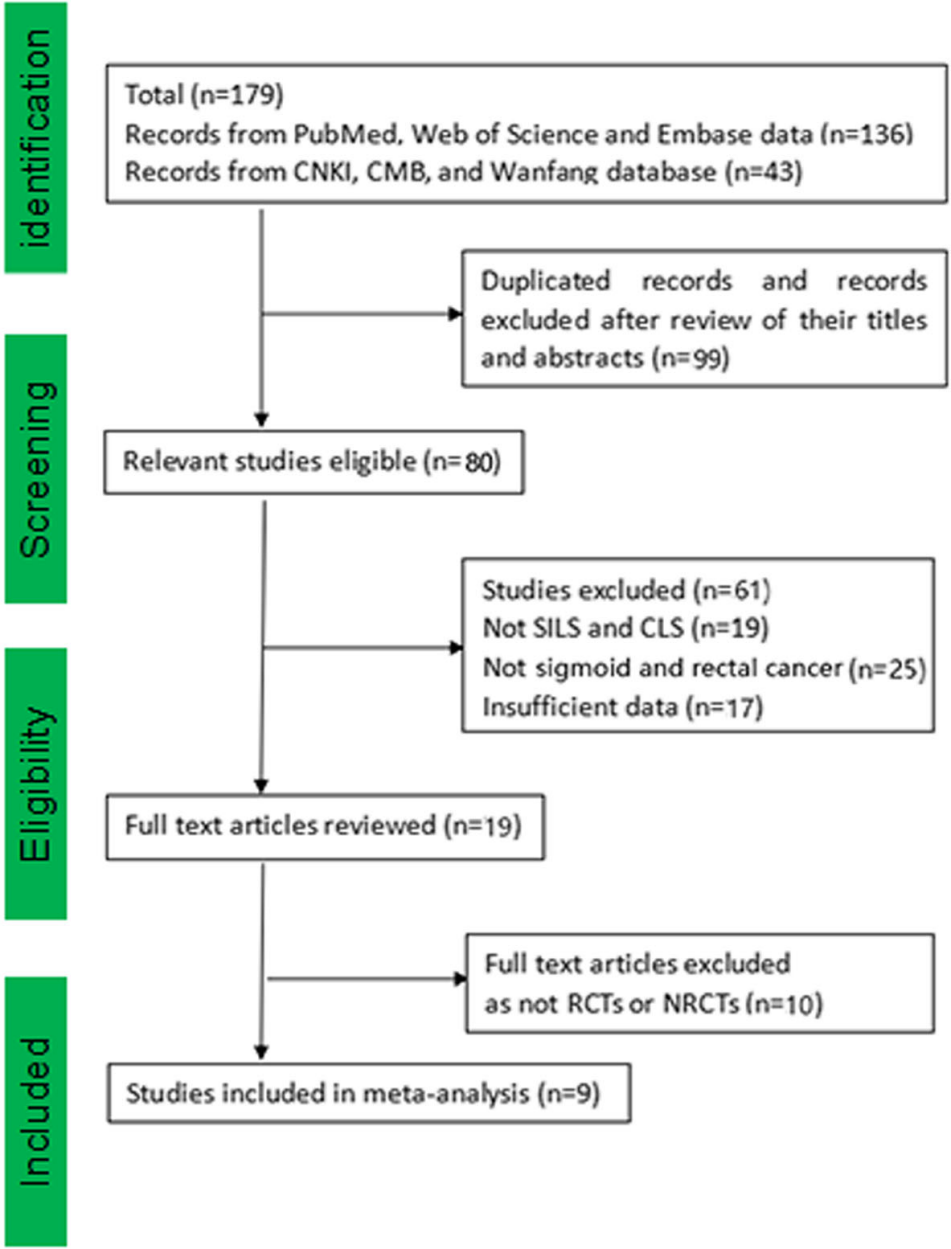
Flowchart of the included studies

We identified 179 publications and found 80 relevant eligible studies. We removed 71 studies (non-SILS or CLS, sigmoid or rectal cancer, RCTs or NRCTs), and finally, nine of these studies met our inclusion criteria, which included two RCTs and seven NRCTs with a total of 829 patients included. Of the nine studies, two studies evaluated sigmoid colon cancer, five studies evaluated rectal cancer, one study evaluated rectosigmoid junction cancer, and one study contained both sigmoid and rectal cancer. This study included three western researches and six eastern researches. This study also contained seven English articles and two Chinese articles. All patients who underwent SILS or CLS were confirmed pathologically for sigmoid colon or rectal cancer [9–17] (Fig. 1).

Of the patients evaluated by these studies, 299 (36.1%) patients underwent SILS and 530 (63.9%) patients underwent CLS. Table 1 shows the baseline characteristics and quality assessment of these nine researches; there was no statistical difference for each study.

**Table 1:** Characteristics of the included studies in the meta-analysis

				Patients (*n*)	BMI		Tumor size (cm)		Sex (M/F)		Age		Tumor location	Score
First author	Year	Study area	Type	SILS/CLS	SILS	CLS	SILS	CLS	SILS	CLS	SILS	CLS		
Liu [7]	2016	China	NRCT	16/32	21.9	22.4	3.6	3.6	13/3	23/9	56.4	55.6	Sigmoid and rectum	5
Hong [8]	2016	China	RCT	43/43	NR	NR	NR	NR	23/20	26/17	52.3	54.1	Rs	3
Bulut [9]	2015	Denmark	RCT	20/20	24	24	2.5	4.0	12/8	12/8	69	73	Rectum	3
Kim [10]	2014	Korea	NRCT	67/49	23.1	23.5	4.3	5.3	44/23	28/21	63.8	61.3	Rectum	7
Levic [11]	2014	Denmark	NRCT	36/194	23.8	25	NR	NR	17/19	133/61	69	68	Rectum	8
Tei [12]	2018	Japan	NRCT	44/49	23.6	22	3.9	4.1	29/15	29/20	66	63	Rectum	8
Kwag [13]	2013	Korea	NRCT	24/48	24.4	24	2.6	3.4	9/15	18/30	59.5	59	Sigmoid	7
Park [14]	2012	Korea	NRCT	37/54	24.7	23.9	NR	NR	21/16	26/28	63.8	59.9	Sigmoid	7
Nerup [15]	2018	Denmark	NRCT	12/41	23.5	25	NR	NR	7/5	13/28	76	69	Rectum	6

*F* female, *M* male, *NR* no record, *RCT* randomized controlled trials, *NRCT* non-randomized controlled trials, *SILS* single-port laparoscopic surgery, *CLS* conventional multi-port laparoscopic surgery, *Rs* rectosigmoid junction cancer

### Quality assessment

According to the modified Jadad rating scale for assessing RCTs, scores between 1 and 3 were considered low quality and scores between 4 and 7 were considered high quality. Due to single blinding and unclear method of randomization, two RCTs got scores of 3 with low quality.

According to NRCT evaluation criteria, scores between 1 and 3 were considered low quality, scores between 4 and 6 were considered moderate quality, and scores between 7 and 9 points were considered high quality. The included NRCTs all had moderate or high quality. The specific scores of RCTs and NRCTs are shown in Table 1.

### Meta-analysis results

#### Intraoperative index

**Fig. 2:**
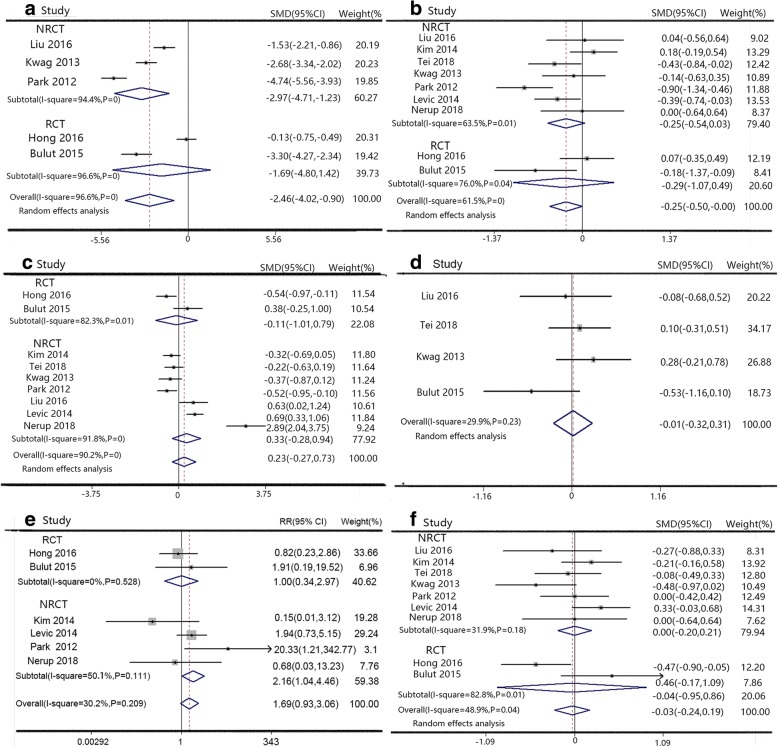
Forest plot of intraoperative outcome. **a** Operation time, **b** incision length, **c** amount of bleeding, **d** conversion rates, **e** lymph node resection, and **f** distal surgical edge (DSE)

The incision length was shorter in SILS than CLS (SMD − 2.46, 95% CI − 4.02 to − 0.90), with large heterogeneity in random effects model (*P* = 0, *I*^2^ = 95.6%, Fig. 2a). SILS had more lymph node resection than CLS in random effects model (SMD − 0.25, 95% CI − 0.50 to − 0.002, *P* = 0, *I*^2^ = 61.5%, Fig. 2b) Two groups had similar results in operative time with CLS (SMD 0.23, 95% CI − 0.27 to 0.73, Fig. 2c), amount of bleeding (SMD − 0.01, 95% CI − 0.32 to 0.31, Fig. 2d), conversion rate (RR 1.69, 95% CI 0.93 to 3.05, Fig. 2e), and distal surgical edge (SMD − 0.03, 95% CI − 0.24 to 0.19, Fig. 2f). All studies had significant heterogeneity in random effects model, except conversion rate without significant heterogeneity in fixed effects model. In subgroup analysis, RCTs had shorter incision length, but higher conversion rate than NRCTs, and other index in RCTs and NRCTs were similar. The detailed values are shown in Table 2.

**Table 2:** Comparison of intraoperative index between SILS and CLS for the included studies

	Operation time (min)		Incision length (cm)		Amount of bleeding (ml)		Conversion		Lymph node resection		Distal surgical edge (cm)	
First author	SILS	CLS	SILS	CLS	SILS	CLS	SILS	CLS	SILS	CLS	SILS	CLS
Liu [7]	126.9 ± 40.3	106.9 ± 26.7	4.8 ± 1.5	6.8 ± 1.2	46.3 ± 61.1	50.3 ± 39.3	NR	NR	21.3 ± 8.1	21 ± 7.5	5.8 ± 2.3	6.6 ± 3.2
Hong [8]	122.3 ± 23.4	137.6 ± 32.4	4.4 ± 3.5	4.8 ± 2.8	NR	NR	4	5	18.2 ± 8.1	17.6 ± 8.9	5.8 ± 1.9	6.8 ± 2.3
Bulut [9]	295 (108–465)	264 (125–421)	4 (2.5–12.5)	13.3 (7–19.5)	33 (0–300)	100 (0–650)	2	1	14 (4–33)	19 (7–33)	3.2 (0.5–7.5)	2.5 (1–6.5)
Kim [10]	277 ± 106	309 ± 93	NR	NR	NR	NR	0	2	23.4 ± 15.3	20.9 ± 12.6	6.9 ± 5.5	5.8 ± 4.9
Levic [11]	295 (108–465)	248 (51–431)	NR	NR	35 (0–400)	100 (0–3142)	5	13	13 (3–33)	16 (1–48)	3 (0.5–7.5)	2.5 (0–9.5)
Tei [12]	198 ± 52.8	210 ± 55	NR	NR	34.5 ± 106	24.8 ± 85	1	0	23 ± 10	28 ± 13	2.97 ± 0.85	3.04 ± 0.88
Kwag [13]	135 ± 28	144 ± 22	3.4 ± 1.1	7.3 ± 1.6	251 ± 50	237 ± 49	0	0	19.6 ± 10.7	20.8 ± 7.7	7.5 ± 2.5	9.2 ± 4
Park [14]	118.1 ± 41.5	140 ± 42.2	3.3 ± 0.9	9.1 ± 1.4	NR	NR	8	0	14.6 ± 6.8	23.4 ± 11.4	5.1 ± 2.5	5.1 ± 2.6
Nerup [15]	316.5 (294–323.3)	269 (236.5–309)	NR	NR	50 (0–200)	150 (62.5–250)	0	2	12 (9–17)	12 (7–15)	4 (3.5–5)	4 (2–5)

#### Postoperative data

**Fig. 3:**
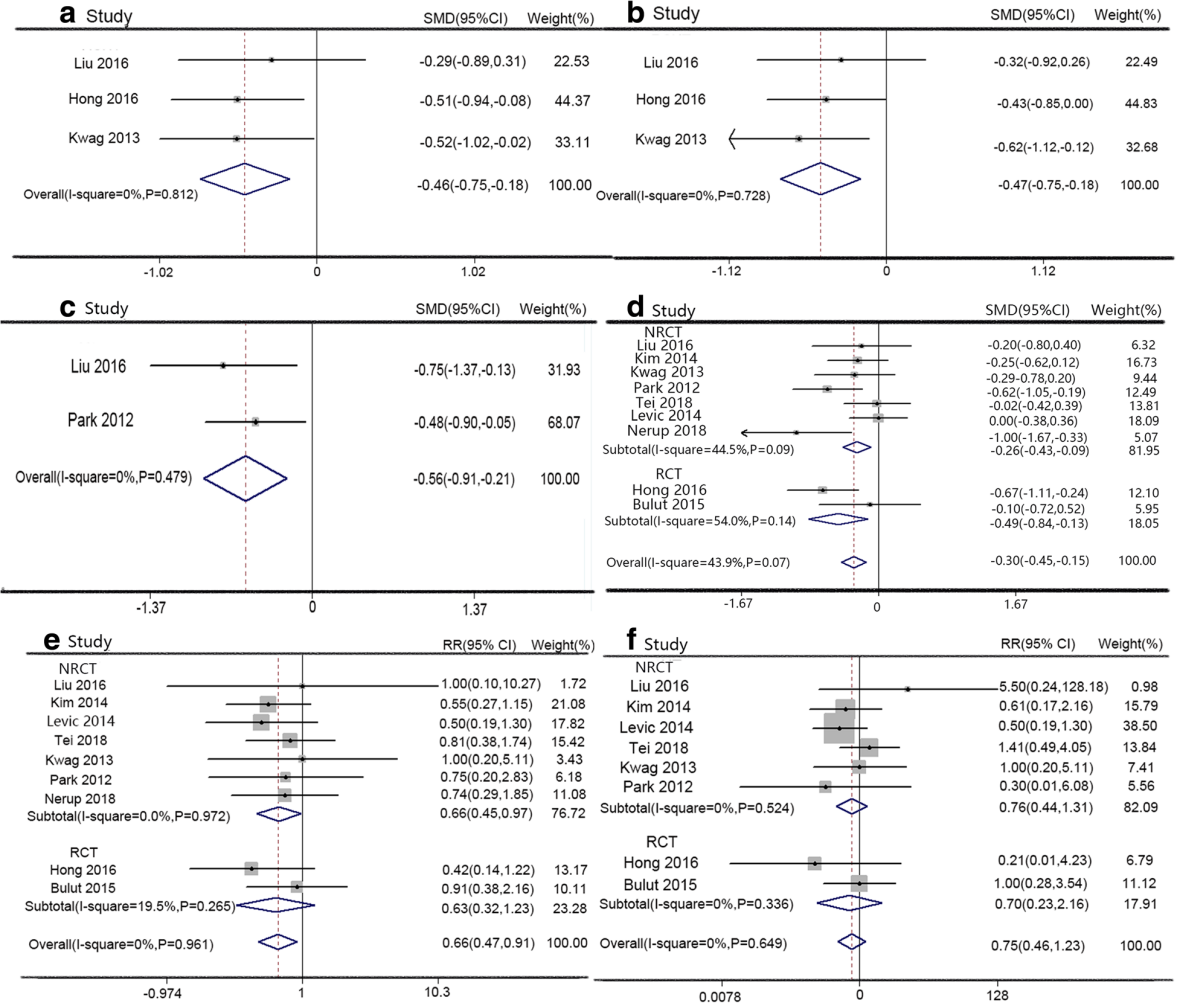
Forest plot of postoperative outcomes. **a** Complication, **b** anastomotic leakage, **c** defecation time, **d** exhaust time, **e** pain score, and **f** hospitalization time

This study showed SILS had obvious advantages over CLS in complication (RR 0.66, 95% CI 0.47 to 0.91, Fig. 3e), defecation time (SMD − 0.46, 95% CI − 0.75 to − 0.18, Fig. 3a), exhaust time (SMD − 0.46, 95% CI − 0.75 to − 0.18, Fig. 3b), pain score (SMD − 0.56, 95% CI − 0.91 to − 0.21, Fig. 3c), and hospitalization time (SMD − 0.30, 95% CI − 0.45 to − 0.15, Fig. 3d). No significant heterogeneity was discovered in two groups except for hospitalization time with high heterogeneity. There was no obvious difference in anastomotic fistula rate between SILS and CLS groups (RR 0.752, 95% CI 0.46 to 1.23, Fig. 3f). SILS mainly contributed to the part of postoperative recovery. The detailed values are shown in Table 3.

**Table 3:** Comparison of postoperative data and follow-up outcomes between SILS and CLS for the included studies

	Complication		Anastomotic fistula		Defection time (days)		Exhaust time (days)		Pain score		Hospitalization time (days)		Readmission		Recurrence		Metastasis	
First author	SILS	CLS	SILS	CLS	SILS	CLS	SILS	CLS	SILS	CLS	SILS	CLS	SILS	CLS	SILS	CLS	SILS	CLS
Liu [7]	1	2	1	0	3.3 ± 0.9	3.6 ± 1.1	2.4 ± 1	2.7 ± 0.9	4.3 ± 1.4	5.2 ± 1.1	8.4 ± 5.3	9.2 ± 3.1	NR	NR	NR	NR	2	2
Hong [8]	4	11	0	2	4.8 ± 2.1	5.9 ± 2.2	2.5 ± 2.3	3.4 ± 1.9	NR	NR	6.7 ± 3.7	8.8 ± 2.4	NR	NR	NR	NR	NR	NR
Bulut [9]	7	8	4	4	NR	NR	NR	NR	NR	NR	7 (3–51)	8 (4–30)	4	1	NR	NR	NR	NR
Kim [10]	10	15	4	5	NR	NR	NR	NR	NR	NR	12.6 ± 11.2	15.3 ± 10.1	NR	NR	NR	NR	NR	NR
Levic [11]	4	49	4	49	NR	NR	NR	NR	NR	NR	7 (3–51)	7 (3–80)	5	22	0	3	2	29
Tei [12]	9	13	6	6	NR	NR	NR	NR	NR	NR	13.5 ± 11.2	13.7 ± 11.6	NR	NR	1	4	1	7
Kwag [13]	2	4	2	4	1.7 ± 0.6	2.2 ± 1.1	2.8 ± 1	3.6 ± 1.4	NR	NR	7.1 ± 3.4	8.1 ± 3.5	NR	NR	NR	NR	NR	NR
Park [14]	3	6	0	2	NR	NR	NR	NR	2.6 ± 1.3	3.4 ± 1.9	5.5 ± 2.3	7.7 ± 4.2	NR	NR	NR	NR	NR	NR
Nerup [15]	4	21	NR	NR	NR	NR	NR	NR	NR	NR	7 (7–9)	8 (7–11.5)	2	5	NR	NR	4	8

*NR* no record

### Follow-up outcomes

**Fig. 4:**
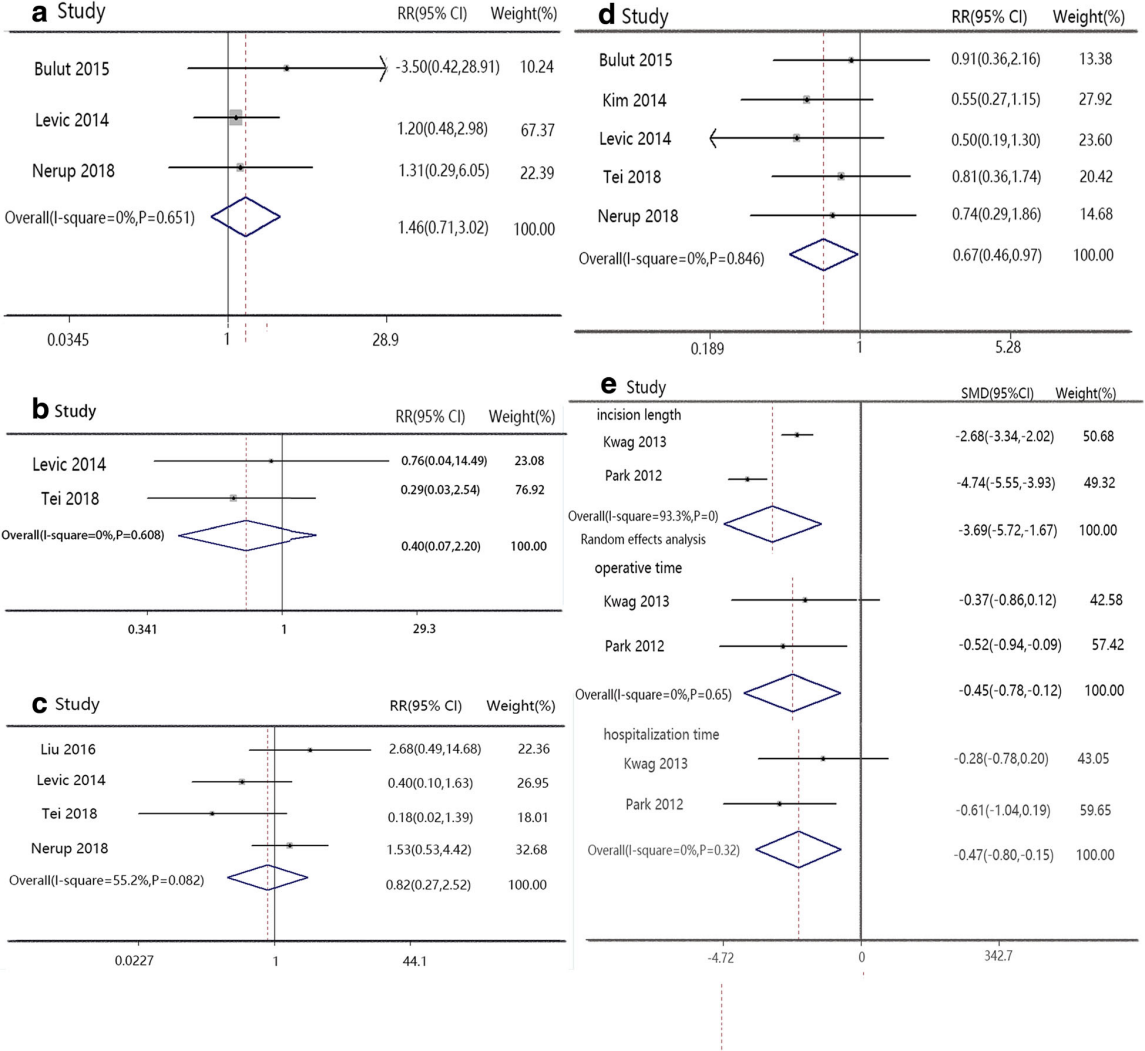
Forest plot of mid-term outcomes. **a** Readmission, **b** local recurrence, **c** metastasis and sigmoid colon cancer versus rectal cancer: **d** complication and **e** incision length, operation time, and hospitalization time

There were no significant differences in readmission (RR 1.46, 95% CI 0.71 to 3.02, Fig. 4a), local recurrence (RR 0.40, 95% CI 0.07 to 2.20, Fig. 4b), and distant metastasis (RR 0.82, 95% CI 0.27 to 2.52, Fig. 4c) between SILS and CLS groups. Readmission and local recurrence used fixed effect model with no significant heterogeneity, while distant metastasis used random effect model with significant heterogeneity. The detailed values are shown in Table 3.

### Subgroup analysis

#### Sigmoid colon cancer versus rectal cancer

For rectal cancer, subgroup analysis showed SILS had a lower complication rate (RR 0.66, 95% CI 0.45 to 0.97, Fig. 4d) than CLS. However, SILS had shorter incision length (SMD − 3.69, 95% CI − 5.72 to − 1.67, Fig. 4e), shorter operative time (SMD − 0.45, 95% CI − 0.78 to − 0.13, Fig. 4e), and shorter hospitalization time (SMD − 0.47, 95% CI − 0.80 to − 0.15, Fig. 4e) than CLS for sigmoid colon cancer patients.

#### Eastern versus western patients

**Fig. 5:**
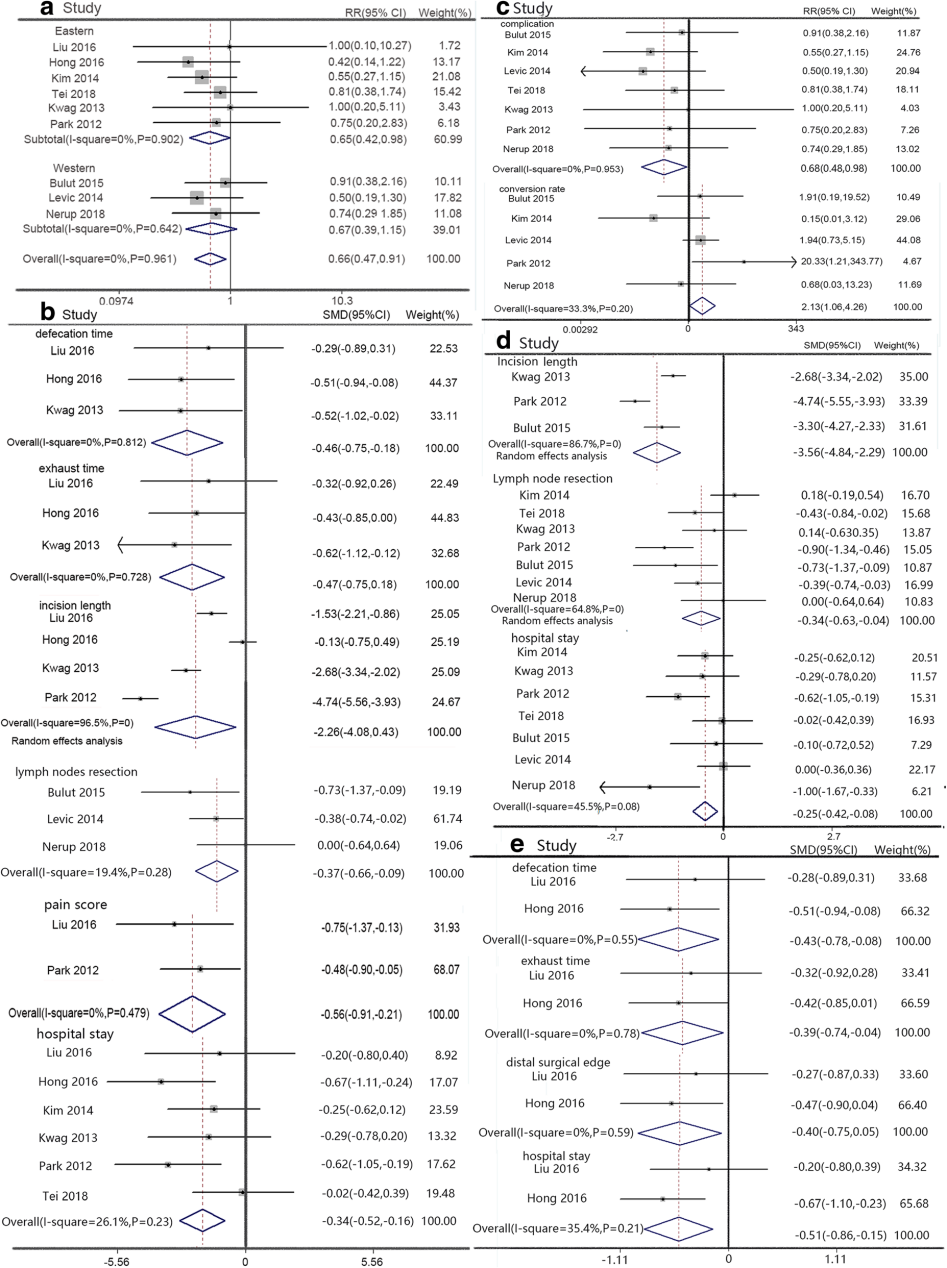
Forest plot of eastern versus western patients. **a** Complication, **b** defecation time, exhaust time, incision length, lymph node resection, pain score, and hospital stay. English versus Chinese studies: **c** complication and conversion; **d** incision length, lymph node resection, and hospital stay; and **e** defecation time, exhaust time, distal surgical edge, and hospital stay

Subgroup analyses related to the region were conducted in further research. In eastern research, SILS had lower complication rate (RR 0.65, 95% CI 0.42 to 0.98, Fig. 5a), faster defecation time (SMD − 0.46, 95% CI − 0.75 to − 0.18, Fig. 5b), faster exhaust time (SMD − 0.47, 95% CI − 0.75 to 0.18, Fig. 5b), and shorter incision length (SMD − 2.26, 95% CI − 4.08 to 0.43, Fig. 5b) than CLS, accompanied with lower pain score (SMD − 0.56, 95% CI − 0.91 to − 0.21, Fig. 5b) and shorter hospital stay (SMD − 0.34, 95% CI − 0.52 to − 0.16, Fig. 5b). But SILS had more lymph node resection (SMD − 0.37, 95% CI − 0.66 to − 0.09, Fig. 5b) than CLS in western research. SILS and CLS had similar results in other indexes.

#### English versus Chinese articles

Seven English articles indicated SILS had a lower complication rate (RR 0.68, 95% CI 0.48 to 0.98, Fig. 5c), more lymph node resection (SMD − 0.34, 95% CI − 0.63 to − 0.04, Fig. 5d), shorter incision length (SMD − 3.56, 95% CI − 4.84 to − 2.29, Fig. 5d), and shorter hospital stay (SMD − 0.25, 95% CI − 0.42 to − 0.08, Fig. 5d), but a higher conversion rate (RR 2.13, 95% CI 1.06 to 4.26, Fig. 5c) compared to CLS. Two Chinese articles contained defecation and exhaust time data and indicated SILS had a shorter defecation time (SMD − 0.43, 95% CI − 0.78 to − 0.08, Fig. 5e) and exhaust time (SMD − 0.39, 95% CI − 0.74 to − 0.04, Fig 5e) than CLS, accompanied with a better distal surgical edge (SMD − 0.40, 95% CI − 0.75 to − 0.05, Fig. 5e) and hospital stay (SMD − 0.51, 95% CI − 0.86 to − 0.15, Fig. 5e).

### Sensitivity analysis

Begg’s correlation test (complication, *P* = 0.639) revealed there was no obvious publication bias. Quality of researches after sensitivity analysis would not impact the final results.

## Discussion

Laparoscopic colorectal surgery has become the trend of the times in modern colorectal surgery. CLS is the traditional laparoscopic surgery; it has become a routine procedure in many hospitals. However, some disadvantages of CLS also existed, such as poor three-dimensional (3D) visualization, limited dexterity of movements, and high conversion rate to open surgery. With the development of medical science, new devices have prompted the wide use of SILS in colorectal surgery. Some studies have demonstrated that SILS is more accurate, effective, and less invasive than CLS in colorectal cancer. However, whether SILS is better than CLS for sigmoid and rectal cancer still remains unclear.

In this meta-analysis, we aimed to collect evidence-based data to compare intraoperative data, postoperative indexes, and short-term follow-up outcomes between SILS with CLS in sigmoid and rectal cancer. We utilized the latest studies to compare outcomes between SILS and CLS for laparoscopic resection in sigmoid colon and rectal cancer; we also carried out subgroup analysis in tumor location, region, and language. Two moderate-quality RCTs and seven moderate- to high-quality NRCTs involving total 829 patients were analyzed for the final results. Our selected studies included moderate sample sizes and provided reliable data to compare the outcomes of the two groups. Among all of the searched articles, two relevant articles were very similar both in background and recruited patients written by Tei et al., so we chose the latest article with long-term follow-up outcomes for our study [18].

The results revealed that SILS had an advantage over CLS in incision length, lymph node resection, complication rate, defecation time, exhaust time, pain score, and hospital stay. No statistical difference was observed in other data. Our results were partially same with that of Li et al. They made a meta-analysis in comparing the effects of SILS with CLS for colorectal cancer and also found that SILS had advantages in incision length, pain score, and hospital stay compared with CLS. Meanwhile, Li et al. also reported SILS with fewer blood transfusion and less blood loss than CLS. Although we did not compare blood transfusion and extra port rate due to incomplete data, SILS still had better outcome than CLS in the above index. Besides, there were some opposite results including lymph node resection, complication rate, operative time, and blood loss between our study and Li et al.’s. In our study, SILS had more lymph node resection and lower complication rate than CLS. We thought that this was due to the different tumor location. Our study focused on sigmoid and rectal cancer and Li et al.’s study focused on colorectal cancer. Different tumor locations could cause more lymph node resection and lower complication rate in our study [19]. Li et al. indicated SILS had less blood loss and longer operative time compared with our study. We think a surgeon could increase operative time due to variation of blood vessels in the right colon [20].

With respect to the conversion rate to open surgery, SILS is similar with CLS. The main reasons could impact conversion rate including obesity, narrow pelvis, important vascular variation, vascular injury, and hypertrophic mesentery [21]. But for sigmoid colon cancer, SILS had a shorter operation time, operative time, and hospital stay than CLS due to good location of sigmoid colon cancer. These results could be affected by the substantial learning curve inherent in performing SILS. The skill of the surgeon could also influence the conversion rate.

The heterogeneity of lower postoperative complication rates especially for rectal cancer in SILS was likely attributable to hospital stay, defecation time, and exhaust time. The complication rate is the main contributor to surgical technique and operative time. SILS with a short incision length could reduce postoperative pain, promote early activities, and cut down the incidence of complication [22]. The heterogeneity of proximal surgical edge might be attributed to variation in surgical skills and experience of surgeons, but with more lymph node resection in SILS. We imaged SILS could cut off enough mesentery to get more lymph nodes, especially with the technique of TME.

Three studies evaluated readmission, two studies evaluated local recurrence, and four studies evaluated distant metastase; SILS and CLS had similar results. Due to the short development time of SILS, lack of clinical data might impact the results of readmission, local recurrence, and distal metastasis. We expect more clinical research to further illuminate the relationship between the groups [23].

In subgroup analysis of region, SILS had better outcomes than CLS, including complication rate, incision length, defecation time, exhaust time, and hospital stay for eastern patients, and SILS had more lymph node resection for western patients than CLS. Western patients had a particularly difficult surgery with high body mass and narrow operation space. Although all surgeries were performed by experienced surgical teams, we still found SILS with a short incision length could reduce the suture time and pain sensation. This finding was the same to some clinical reports [24]. The benefits of minimally invasive surgery could be reflected by incision length, defecation time, exhaust time, and hospital stay [25].

In the subgroup analysis of language, seven English articles had indicated SILS have better results of complication, incision length, lymph node resection, and hospital stay than CLS except for conversion rate. However, two Chinese articles supplied additional data of superior defecation time and exhaust time, accompanied with better distal surgical edge and shorter hospital stay. English articles included more patients’ data than Chinese articles, but Chinese articles added some available data of intestinal movement.

The results of the article could be subjected to some interference due to several limitations. First, nine studies with only a modest number of patients were a limitation that might affect the outcomes and induce bias. Only two RCTs had been published on this subject, while seven retrospective studies had been published, which were not the highest quality of evidence. Second, although the majority of the assessed outcomes across all papers had no dramatic conflicts in the findings between units, variation between different units could influence the outcomes. Third, SILS technique which is not yet popular due to its long learning curve and high cost could affect the results. Additionally, insufficient postoperative follow-up time might also produce a performance bias. In the near future, more large-scale RCTs with complete follow-up data will emerge to reveal the clinical and prognostic effects of SILS [26]. All countries should invest a great deal of financial and material resources to promote SILS for colorectal surgery. With the improving of the equipment, the SILS port could hold more holes which make it easier to contain more forceps to accelerate the operation.

Our meta-analysis provided current information on the role of SILS compared with CLS. We incorporated research into strict standards and used a number of methods to ensure the quality of the included studies. We used Begg’s test to evaluate publication bias. Our study focused on sigmoid colon and rectal cancer and minimized bias for a broad range of colorectal surgery.

## Conclusions

In conclusion, this study confirmed the feasibility and compared the advantages and disadvantages of two techniques. SILS had some advantages, such as shorter hospital stay, smaller incision length, more rapid time to return to bowel function, slighter pain score, and a lower complication rate. SILS and CLS had several similar clinical outcomes, such as blood loss, rate of conversion to open surgery, anastomotic fistula rate, readmission, local recurrence, and distal metastases. With the continuing development of professional technology, future evidence in long-term outcomes could promote widespread use of SILS for sigmoid colon and rectal cancer.

## Abbreviations

BMI: Body mass index
CLS: Conventional multi-port laparoscopic surgery
HR: Hazard ratios
NRCT: Non-randomized controlled trials
OR: Odds ratio
OS: Overall survival
RCTs: Randomized clinical trails
SD: Standard deviation
SILS: Single-port laparoscopic surgery
WMD: Weighted mean difference
